# Cyclooxygenase-2 Expression Is a Predictive Marker for Late Recurrence in Colorectal Cancer

**DOI:** 10.1155/2018/7968149

**Published:** 2018-06-24

**Authors:** Sung Hoo Kim, Byung Kyu Ahn, Seung Sam Paik, Kang Hong Lee

**Affiliations:** ^1^Department of Surgery, Hanyang University College of Medicine, Seoul, Republic of Korea; ^2^Department of Pathology, Hanyang University College of Medicine, Seoul, Republic of Korea

## Abstract

**Introduction:**

Cyclooxygenase-2 (COX-2) expression is elevated in colorectal cancer (CRC). However, data about the relation between COX-2 expression and the impact on the biologic behavior of recurrent disease are inconclusive as yet. The aim of this study is to investigate the relationship between the status of COX-2 expression in the primary CRC and the characteristics of recurrence after curative resection of stage I to III CRC.

**Materials and Methods:**

Ninety-eight patients with recurrence in 376 CRC patients, who underwent curative surgery between January 1991 and August 2001, were retrospectively assessed. Immunohistochemical staining, performed for the presence of COX-2 on tissue microarrays, was analyzed.

**Results:**

Forty-six patients showed elevated COX-2 expression, and 52 patients did not. The mean time to recurrence was significantly longer in the positive group than in the negative group (34.1 months ± 30.0 versus 21.9 months ± 17.4; *P* = 0.019). Positive COX-2 expression was correlated with late recurrence (>3 years after surgery) [43.5% versus 13.5%; *P* = 0.001]. In multivariate analysis, COX-2 expression was an independent factor associated with late recurrence (OR 4.656; 95% CI, 1.696 to 12.779; *P* = 0.003). Recurrence pattern and postrecurrence survival were not different between the two groups.

**Conclusions:**

Elevated COX-2 expression in itself is not a prognostic factor, but COX-2 expression in tumor tissue may be an independent predictive marker of late recurrence for patients with stage I to III CRC.

## 1. Introduction

The mainstay of colorectal cancer (CRC) treatment is curative resection, and tumor recurrence is a major concern after surgery. There have been several attempts to identify molecular markers that can predict recurrence and survival rates, but still, none is approved for clinical application.

Cyclooxygenase-2 (COX-2) is a rate-limiting enzyme involved in the conversion of arachidonic acid to prostaglandins and thromboxanes. These products play crucial roles in cell proliferation, immune response, angiogenesis, and inflammatory reaction, which may involve tumor development and progression [1, 2]. Previous studies have reported that COX-2 overexpression is detected in colorectal, gastric, breast, pulmonary, esophageal, and pancreatic cancer [3–7]. Increased COX-2 gene expression has been reported in human colorectal adenocarcinoma and in carcinogen-induced rat colonic tumors [8–12]. However, the molecular mechanisms by which COX-2 contributes to CRC progression and metastasis remain unclear. In addition, it remains controversial whether COX-2 expression is a prognostic factor for the survival of CRC patients or not [13–15].

The aim of this study is to investigate the relationship between the status of COX-2 expression and the characteristics of recurrent disease in stage I–III CRC patients after curative resection.

## 2. Methods

### 2.1. Selection of Patients

We retrospectively reviewed, between January 1991 and August 2001, 492 patients with the diagnosis of CRC and treated in one tertiary care center. 44 patients were excluded by the inclusion criteria. The inclusion criteria were stage I–III patients with curative resection (R_0_). Patients with (a) distant metastasis (*n* = 29), (b) incomplete resection (*n* = 2), and (c) metachronous cancers (*n* = 3) were excluded. The patients who expired due to other causes (*n* = 10) were also excluded. As a result, formalin-fixed paraffin-embedded samples from 376 patients were available. Patients with lymph node-positive disease received 5-FU-based adjuvant chemotherapy, and none of the patients received preoperative chemotherapy or radiotherapy. The median follow-up period was 56 months (range, 3 to 192 months). Of the 376 patients, COX-2 expression was elevated in 211 patients (56.0%, 211 patients), and the overall recurrence rate was 26.0% (98/376 patients).

### 2.2. Tissue Microarrays (TMAs)

After the histological examination of H&E-stained samples by an experienced pathologist, parts containing a high proportion of tumor cells were assembled. TMAs were constructed with a tissue arrayer (AccuMac Arrayer, ISU ABXIS Co. Ltd., Seoul, Korea). The assembled TMAs were held in an X-Y position guide with 1 mm increments between individual samples and a 3 mm punch-depth stop device. Briefly, this instrument was utilized to make holes in a recipient block with defined array cores, and a solid stylet, which fitted the needle closely, was used to transfer the tissue cores into the recipient block. Due to the limited size of representative areas of the tumors, triplicate 1 mm diameter tissue cores were made from each donor block.

### 2.3. Immunohistochemical Staining

We obtained multiple 4 *μ*m cut sections using a Leica microtome in immunohistochemical staining. The obtained sections were shifted to adhesive-coated slides. Dewaxing was performed with the TMA slides by heating at 55°C for 30 min and by three washes, of 5 min each, with xylene. Rehydration was done with the tissues by 5 min washes in 100%, 90%, and 70% ethanol and phosphate-buffered saline (PBS). Antigen was retrieved by heating the samples for 4 min 20 s in a microwave at full power in 250 ml 10 mM sodium citrate (pH 6.0). Endogenous peroxidase activity was blocked with 0.3% hydrogen peroxidase for 20 min. The sections were incubated with primary goat polyclonal anti-COX-2 antibody (N-20; Santa Cruz Biotechnology, Santa Cruz, CA, USA) diluted 1 : 100 in goat serum at room temperature for 1 h. After three washes of 2 min each with PBS, the sections were incubated with biotinylated anti-goat secondary antibody for 30 min (DAKO, Carpinteria, CA, USA). After three further washes with PBS, horseradish peroxidase streptavidin (DAKO) was added to the section for 30 min, followed by another three washes. The samples were developed for 1 min with 3,3′-diaminobenzidine substrate (Vector Laboratories, Burlington, Ontario, Canada) and counterstained with Mayer's hematoxylin. They were dehydrated according to standard procedures and closed with coverslips.

### 2.4. Interpretation of COX-2 Expression

**Figure 1:**
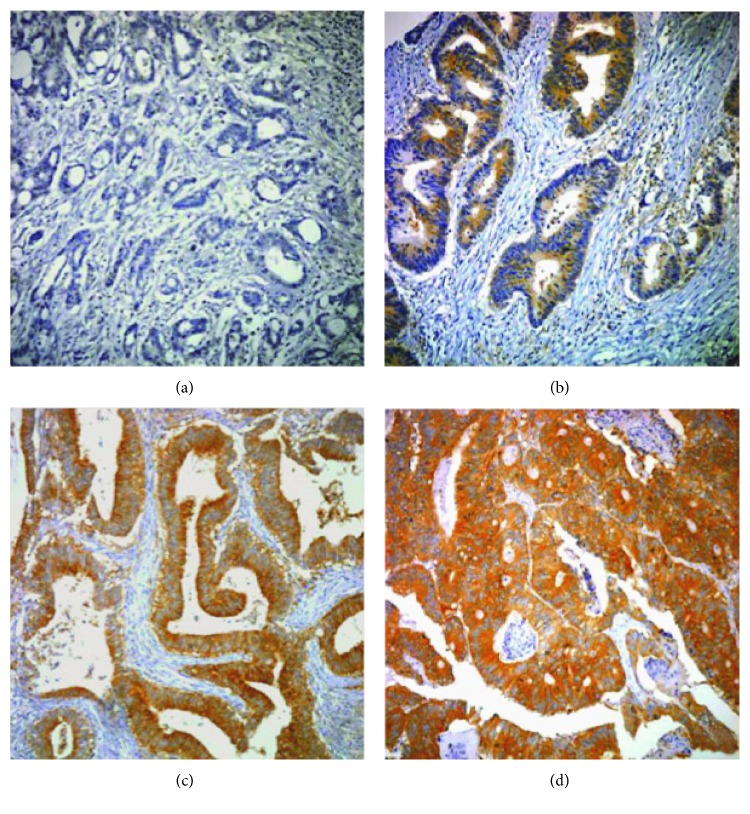
The microphotographs of COX-2 immunostaining by intensity in colorectal cancer: (a) negative, (b) weak, (c) moderate, and (d) strong.

COX-2 expression was interpreted independently by two experienced pathologists (SS Paik and SH Jang) on the basis of staining intensity and extent. Three punches per case were evaluated and considered a whole. Staining intensity was scored as 0 to 3 (0 = negative; 1 = weak; 2 = moderate; and 3 = strong) (Figure 1). Staining extent was scored as 0 to 4 based on the percentage of positive-stained cells (0 = 0%; 1 = 1–25%; 2 = 26–50%; 3 = 51–75%; and 4 = 76–100%). The final staining score was determined with a sum of the intensity and extent score. We divided all cases into four expression groups based on their sum of scores (0 = negative; 1–3 = low; 4-5 = moderate; and 6-7 = high). If the sum of scores was ≥4, we classified the cases as elevated COX-2 expression (positive). If the sum of scores was ≤3, we classified the cases as COX-2 negative. When there was a disagreement between the two pathologists, reinvestigation of the slide was performed with a multiheaded microscope and the final agreement was achieved.

### 2.5. Statistical Analysis

Statistical analysis was conducted with SPSS ver. 19.0 (SPSS Inc., Chicago, IL, USA). The chi-square test and Student's *t*-test were used to examine the association between COX-2 expression and clinical and pathological features including age, gender, tumor location, tumor size, gross type, cell type, differentiation, lymphatic invasion, vascular invasion, T category, N category, AJCC stage, and time to recurrence. Survival curves were constructed using the Kaplan-Meier method, and survival differences were analyzed by the log-rank test. *P* values < 0.05 were considered statistically significant.

## 3. Results

### 3.1. COX-2 Expression Is Related with Time to Recurrence

The clinical and pathological characteristics of the 376 patients are described in Table 1 according to the COX-2 expression status. In univariate analysis, the COX-2-positive group was different from the COX-2-negative group in terms of differentiation, lymphatic invasion, N category, AJCC stage, and recurrence rate. The clinical and pathologic findings of the 98 patients with tumor recurrence are included in Table 2. There were no statistically significant differences in terms of age, gender, tumor location, tumor size, gross type, cell type, differentiation, lymphatic invasion, vascular invasion, T category, N category, or AJCC stage between the two groups except for the time to recurrence. This was significantly longer in the COX-2-positive group than in the COX-2-negative group (34.1 months ± 30.0 versus 21.9 months ± 17.4, *P* = 0.019).

71 out of 98 patients with recurrence experienced early recurrence (72.4%), and 27 patients had late recurrence (27.6%). In univariate analysis, lymphatic invasion and positive COX-2 expression were significantly different between the two groups (*P* = 0.012 and *P* = 0.001, resp.) (Table 3). Positive lymphatic invasion was significantly correlated with early recurrence (*P* = 0.012), while positive COX-2 expression was significantly related with late recurrence (*P* = 0.001). Multivariate analysis revealed that lymphatic invasion was an independent factor for the early recurrence (OR 0.309; 95% CI, 0.103 to 0.924; *P* = 0.036), and positive COX-2 expression was an independent factor for the late recurrence (OR 4.656; 95% CI, 1.696 to 12.779; *P* = 0.003) (Table 3).

### 3.2. Recurrence Patterns and Postrecurrence Survival according to the COX-2 Expression Status

**Figure 2:**
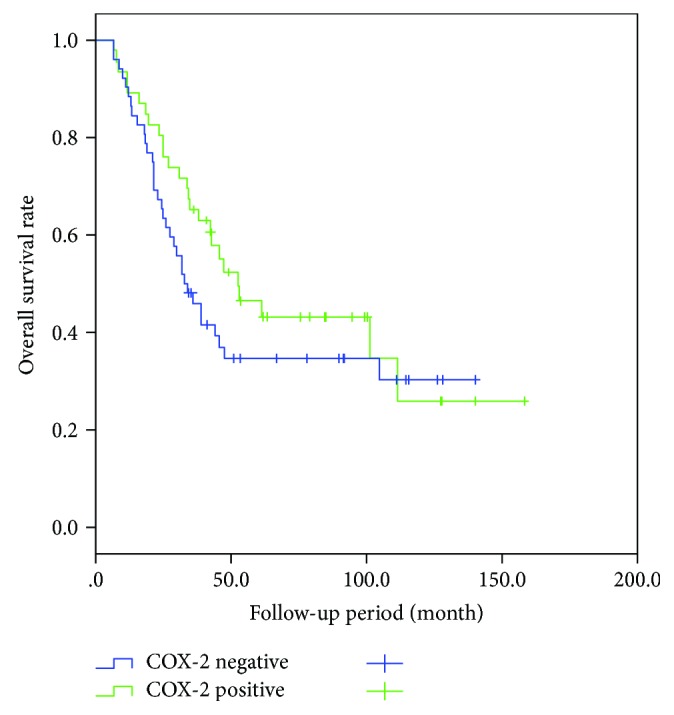
Postrecurrence survival was not significantly different according to the COX-2 status of the 98 patients with recurrence (*P* = 0.230).

Thirty-two (8.5%) of the 376 patients experienced local recurrence, and 66 (17.5%) had distant metastasis. The most common site of distant metastasis was the liver (*n* = 25, 6.6%) followed by the lung (*n* = 19, 5.1%). The patterns of recurrence in the positive and the negative COX-2 expression groups were not different (Table 4). In the 98 patients with recurrence, there was no relation on postrecurrence survival according to the COX-2 expression status (Figure 2).

## 4. Discussion

Our results suggest that COX-2 expression in CRC is associated with late recurrence (>3 years after surgery) during the postsurgery follow-up period, which may not mean that COX-2 expression prevents early recurrence. In this study, lymphatic invasion was a significant factor for the early recurrence but COX-2 expression was a significant factor for the late recurrence. We assumed that positive COX-2 expression do not prevent early recurrence but induce late recurrence. Maybe there are other mechanisms which contribute to recurrence in the positive COX-2 expression group different from the lymphatic invasion group. A number of mechanisms may be involved in the process of late recurrence. COX-2 overexpression increases the migration and proliferation of intestinal epithelial cell and inhibits programmed cell death, so prolonging the survival of abnormal cells [16]. Interestingly, another study found that COX-2 overexpression was correlated with elevated intracellular telomerase and reduced apoptosis [17]. In nonsmall cell lung cancer, COX-2 overexpression has been shown to stabilize survivin, an inhibitor of apoptosis [18]. In breast cancer, the presence of cytoplasmic survivin positively correlates with COX-2 expression [19]. COX-2 and survivin are overexpressed and positively correlated in endometrial adenocarcinoma [20]. Although our data do not have a bearing on molecular mechanisms, we suspect that the relation between COX-2 overexpression and late recurrence may be due to a decreased rate of apoptosis of surviving tumor cells. We suspect that surviving tumor cells in the stromal compartment may grow and migrate to other organs, and time may be required for surviving tumor cells to acquire resistance to adjuvant treatment.

Some studies have suggested that elevated COX-2 expression of CRC patients is related with reduced survival [15, 21]. However, others found that the elevated expression of COX-2 protein had no significant impact on disease-specific survival and overall survival in CRC patients [22, 23]. We observed no significant postrecurrence survival difference according to COX-2 expression status. Thus, COX-2 expression is not likely a prognostic factor for postrecurrence in CRC.

After curative surgery, CRC patients with positive COX-2 expression have an increased probability of late tumor recurrence based on the result of this study. Therefore, the positive COX-2 patients should be considered candidates for more frequent testing after 3 years of follow-up and extend follow-up period longer than 5 years after surgery. In protocols for postsurgery surveillance, there is a tendency for the frequency of follow-up and testing to be reduced after 3 years. We suggest that since COX-2 expression may be a marker for late recurrence, the frequency of follow-up and testing should not be reduced after 3 years. Furthermore, suspending follow-up after 5 years from the initial operation may be inappropriate especially in COX-2-positive patients. A further prospective randomized study is required to identify optimal surveillance methods and follow-up intervals.

As smoking habit and body mass index may modify the risk of CRC in COX-2 genotype, this bias could affect our conclusions regarding the predictive marker [24]. A further well-designed, large, sample-sized study is mandatory.

A limitation of this study was its retrospective design, which is subject to selection bias. Also, the cases were all from a single institution. As no molecular biological study was performed, it was not clear how COX-2 expression contributed to late recurrence. But it is meaningful that the study revealed a novel finding about the relationship between elevated COX-2 expression and late recurrence of CRC: we were able to demonstrate the possibility of COX-2 expression as a biologic marker predicting late recurrence in CRC patients.

## 5. Conclusions

Elevated COX-2 expression in itself is not a prognostic factor, but COX-2 expression in tumor tissue may be an independent predictive marker of late recurrence for patients with stage I to III CRC. A further well-designed study is required to demonstrate the regulatory mechanism of COX-2 expression on CRC recurrence.

## Figures and Tables

**Table 1:** Clinical and pathological characteristics according to COX-2 expression in 376 patients with R0 resection.

Variable	Total patient (*n* = 376)	COX-2 negative (*n* = 165)	COX-2 positive (*n* = 211)	*P* value
Age (yr)				0.496
<70	311 (82.7%)	134 (81.2%)	177 (83.9%)	
≥70	65 (17.3%)	31 (18.8%)	34 (16.1%)	
Gender				0.436
Male	208 (55.3%)	95 (57.6%)	113 (53.6%)	
Female	168 (44.7%)	70 (42.4%)	98 (46.4%)	
Tumor location				0.209
Right colon	82 (21.8%)	34 (20.6%)	48 (22.7%)	
Left colon	113 (30.1%)	43 (26.1%)	70 (33.2%)	
Rectum	181 (48.1%)	88 (53.3%)	93 (44.1%)	
Tumor size				0.961
<5 cm	118 (31.4%)	52 (31.5%)	66 (31.3%)	
≥5 cm	258 (68.6%)	113 (68.5%)	145 (68.7%)	
Gross type				0.297
Fungating	180 (44.9%)	83 (50.3%)	97 (46.0%)	
Infiltrative	170 (46.9%)	69 (41.8%)	101 (47.8%)	
Unknown	26 (8.2%)	13 (7.9%)	13 (6.2%)	
Cell type				0.303
Nonmucinous	356 (94.7%)	154 (93.3%)	202 (95.7%)	
Mucinous	20 (5.3%)	11 (6.7%)	9 (4.3%)	
Differentiation				0.032
WD	10 (2.7%)	5 (3.0%)	5 (2.4%)	
MD	301 (80.1%)	141 (85.5%)	160 (75.8%)	
PD	65 (17.3%)	19 (11.5%)	46 (21.8%)	
Lymphatic invasion				0.004
Absent	180 (47.9%)	65 (39.4%)	115 (54.5%)	
Present	196 (52.1%)	100 (60.6%)	96 (45.5%)	
Vascular invasion				0.634
Absent	372 (98.9%)	164 (99.4%)	208 (98.6%)	
Present	4 (1.1%)	1 (0.6%)	3 (1.4%)	
T category				0.089
T1	7 (1.9%)	1 (0.6%)	6 (2.8%)	
T2	32 (8.5%)	9 (5.5%)	23 (10.9%)	
T3	330 (87.8%)	152 (92.1%)	178 (84.4%)	
T4	7 (1.9%)	3 (1.8%)	4 (1.9%)	
N category				0.019
N0	185 (49.2%)	69 (41.8%)	116 (55.0%)	
N1	90 (23.9%)	41 (24.8%)	49 (23.2%)	
N2	101 (26.9%)	55 (33.3%)	46 (21.8%)	
Stage				0.003
I	31 (8.2%)	7 (4.2%)	24 (11.4%)	
II	158 (42.0%)	62 (37.6%)	96 (45.5%)	
III	187 (49.7%)	96 (58.2%)	91 (43.1%)	
Recurrence				0.001
Early (≤3 yrs)	71 (18.9%)	45 (27.4%)	26 (12.3%)	
Late (>3 yrs)	27 (7.2%)	7 (4.3%)	20 (9.4%)	
No	278 (73.9%)	112 (67.9%)	166 (78.7%)	

**Table 2:** Clinical and pathological characteristics according to COX-2 expression in patients with recurrence.

Variable	Total patient (*n* = 98)	COX-2 negative (*n* = 52)	COX-2 positive (*n* = 46)	*P* value
Age (yr)				0.200
<70	80 (81.6%)	40 (76.9%)	40 (87.0%)	
≥70	18 (18.4%)	12 (23.1%)	6 (13.0%)	
Gender				0.267
Male	56 (57.1%)	27 (51.9%)	29 (63.0%)	
Female	42 (42.9%)	25 (48.1%)	17 (37.0%)	
Tumor location				0.725
Right colon	25 (25.5%)	13 (25.0%)	12 (26.1%)	
Left colon	16 (16.3%)	9 (17.3%)	7 (15.2%)	
Rectum	57 (58.2%)	30 (57.7%)	26 (58.7%)	
Tumor size				0.286
<5 cm	33 (33.7%)	20 (38.5%)	13 (28.3%)	
≥5 cm	65 (66.3%)	32 (61.5%)	33 (71.7%)	
Gross type				0.680
Fungating	44 (44.9%)	22 (42.3%)	22 (47.8%)	
Infiltrative	46 (46.9%)	25 (48.0%)	21 (45.6%)	
Unknown	8 (8.2%)	5 (9.6%)	3 (6.5%)	
Cell type				1.000
Nonmucinous	93 (94.9%)	49 (94.2%)	44 (95.7%)	
Mucinous	5 (5.1%)	3 (5.8%)	2 (4.3%)	
Differentiation				0.291
WD	4 (4.1%)	2 (3.8%)	2 (4.3%)	
MD	75 (76.5%)	43 (82.7%)	32 (69.6%)	
PD	19 (19.4%)	7 (13.5%)	12 (26.1%)	
Lymphatic invasion				0.190
Absent	20 (20.4%)	8 (15.4%)	12 (26.1%)	
Present	78 (79.6%)	44 (84.6%)	34 (73.9%)	
Vascular invasion				1.000
Absent	98 (100%)	52 (100%)	46 (100%)	
Present	0 (0%)	0 (0%)	0 (0%)	
T category				0.840
T1	0 (0%)	0 (0%)	0 (0%)	
T2	3 (3.1%)	1 (1.9%)	2 (4.3%)	
T3	92 (93.9%)	49 (94.2%)	43 (93.5%)	
T4	3 (3.1%)	2 (3.8%)	1 (2.2%)	
N category				0.479
N0	22 (22.4%)	11 (21.2%)	11 (23.9%)	
N1	27 (27.6%)	17 (32.7%)	10 (21.7%)	
N2	49 (50.0%)	24 (46.2%)	25 (54.3%)	
Stage				0.713
I	1 (1%)	0 (0%)	1 (2.2%)	
II	22 (22.4%)	11 (21.2%)	11 (23.9%)	
III	75 (76.6%)	41 (78.8%)	34 (73.9%)	
Time to recurrence (month)	27.6 ± 24.8	21.9 ± 17.4	34.1 ± 30.0	0.019
Recurrence type				0.001
Early (≤3 yrs)	71 (72.4%)	45 (86.5%)	26 (56.5%)	
Late (>3 yrs)	27 (27.6%)	7 (13.5%)	20 (43.5%)	

**Table 3:** Univariate and multivariate analysis of independent risk factors associated with late recurrence.

Variable	Early recurrence (<3 yr) (*n* = 71)	Late recurrence (≥3 yr) (*n* = 27)	Univariate analysis (*P* value)	Multivariate analysis (OR, 95% CI, *P* value)
Age (yr)			0.382	
<70	56 (78.9%)	24 (88.9%)		
≥70	15 (21.1%)	3 (11.1%)		0.726 (0.174–3.024) 0.660
Gender			0.473	
Male	39 (54.9%)	17 (63.0%)		0.996 (0.361–2.747) 0.993
Female	32 (45.1%)	10 (37.0%)		
Tumor location			1.000	
Right colon	18 (25.3%)	7 (25.9%)		
Left colon	12 (16.9%)	4 (14.8%)		
Rectum	41 (57.7%)	16 (59.3%)		
Tumor size			0.965	
<5 cm	24 (33.8%)	9 (33.3%)		
≥5 cm	47 (66.2%)	18 (66.7%)		
Gross type			0.280	
Fungating	30 (42.3%)	14 (51.9%)		
Infiltrative	36 (50.7%)	10 (37.0%)		
Unknown	5 (7.0%)	3 (11.1%)		
Cell type			0.614	
Nonmucinous	68 (95.8%)	25 (92.6%)		
Mucinous	3 (4.2%)	2 (7.4%)		
Differentiation			0.818	
WD	3 (4.2%)	1 (3.7%)		
MD	53 (74.6%)	22 (81.5%)		
PD	15 (21.1%)	4 (14.8%)		
Lymphatic invasion			0.012	
Absent	10 (14.1%)	10 (37.0%)		
Present	61 (85.9%)	17 (63.0%)		0.309 (0.103–0.924) 0.036
Vascular invasion			1.000	
Absent	71 (100%)	27 (100%)		
Present	0 (0%)	0 (0%)		
T category			0.193	
T1	0 (0%)	0 (0%)		
T2	1 (1.4%)	2 (7.4%)		
T3	67 (94.4%)	25 (92.6%)		
T4	3 (4.2%)	0 (0%)		
N category			0.102	
N0	12 (16.9%)	10 (37.0%)		
N1	21 (29.6%)	6 (22.3%)		
N2	38 (53.5%)	11 (40.7%)		
Stage			0.051	
I	0 (0%)	1 (3.7%)		
II	13 (18.3%)	9 (33.3%)		
III	58 (81.7%)	17 (63.0%)		1.315 (0.256–6.753) 0.743
COX-2 expression			0.001	
Negative	45 (63.4%)	7 (25.9%)		
Positive	26 (36.6%)	20 (74.1%)		4.656 (1.696–12.779) 0.003

**Table 4:** Recurrence pattern according to the status of COX-2 expression in the 98 patients with recurrence.

	COX-2 negative (*n* = 52)	COX-2 positive (*n* = 46)	*P* value
			0.256
Local recurrence	14 (26.9%)	18 (39.1%)	
Liver	13 (25.0%)	12 (26.1%)	
Lung	9 (17.3%)	10 (21.7%)	
Peritoneal seeding	5 (9.6%)	3 (6.5%)	
Others (brain, bone, skin)	11 (21.2%)	3 (6.5%)	
